# Smoking, but Not Other Environmental Exposures, Increases Risk for Advanced Brain Aging

**DOI:** 10.1093/geroni/igaa057.898

**Published:** 2020-12-16

**Authors:** Nathan Whitsel, Carol Franz, William Kremen

**Affiliations:** 1 UCSD, San Diego, California, United States; 2 University of California San Diego, La Jolla, California, United States; 3 University of California San Diego, San Diego, California, United States

## Abstract

Exposure to harmful substances and chemicals such as tobacco smoke, chemicals (e.g., herbicides, pesticides, Agent Orange) and metal dust has been associated with increased risk of developing cancer, cardiovascular disease, and other diseases that contribute to shorter life expectancy. Associations with brain health in relation to these exposures are less well studied. We examined the relationship between brain health and prolonged exposure to different harmful substances in 498 male participants average age 68 (range 61 to 73) from the Vietnam Era Twin Study of Aging (VETSA). For self-reported tobacco smoke, herbicides/pesticides, and metal dust we created three groups reflecting recency of exposure (current/former/never). For Agent Orange we examined two exposure groups (ever/never). Brain health, defined as predicted brain age (PBAD), was evaluated by applying Brain-Age Regression Analysis and Computation Utility software (BARACUS) to magnetic resonance images collected at age 68. Tobacco smoking (r=-0.15, p=0.0004 ) was significantly correlated with PBAD and remained significant (F=5.56, p=0.005) in multivariate analyses adjusted for age, socioeconomic status (SES), age 20 general cognitive ability, and non-independence of twins within pairs. Never smokers had significantly younger brains than current or former smokers. PBAD did not differ for current versus former smokers. In other analyses, more advanced PBAD was associated with non-amnestic MCI. In this sample, tobacco smoking had the strongest relationship with overall brain health in late midlife compared with other types of environmental exposures, reinforcing its role in pathological aging and its importance as a public health priority.

